# The Mediterranean Diet Reduces the Risk and Mortality of the Prostate Cancer: A Narrative Review

**DOI:** 10.3389/fnut.2017.00038

**Published:** 2017-08-24

**Authors:** Cristiano Capurso, Gianluigi Vendemiale

**Affiliations:** ^1^Department of Medical and Surgical Sciences, University of Foggia, Foggia, Italy

**Keywords:** prostate cancer, obesity, nutrition, Mediterranean diet, olive oil

## Abstract

Prostate cancer is the second most common cancer in the world among men, and is the fifth most common cause of cancer death among men. The aim of our review was to analyze observational and case–control studies to point out the effects of overweight and diets components on the cancer risk, particularly on risk of prostate cancer, and the effect of the Mediterranean diet (MD) on the reduction of risk and mortality of prostate cancer. It is known that incidence and progression of cancer is multifactorial. Cancer of the large bowel, breast, endometrium, and prostate are due also to a high body mass index and to high consumption of high carcinogenic dietary factors, as red and processed meat or saturated fats rich foods, and to a low consumption of vegetables and fruits. Previous meta-analysis suggested that high adherence to diet model based on the traditional MD pattern gives a significant protection from incidence and mortality of cancer of all types. The main component of the MD is olive oil, consumed in high amount by Mediterranean basin populations. In addition, phenolic compounds exert some strong chemo-preventive effects, which are due to several mechanisms, including both antioxidant effects and actions on cancer cell signaling and cell cycle progression and proliferation. The protective effect of the MD against the prostate cancer is also due to the high consumption of tomato sauce. Lycopene is the most relevant functional component in tomatoes; after activating by the cooking of tomato sauce, it exerts antioxidant properties by acting in the modulation of downregulation mechanisms of the inflammatory response. MD, therefore, represents a healthy dietary pattern in the context of a healthy lifestyle habits. In conclusion, our narrative review allows us to reaffirm how nutritional factors play an important role in cancer initiation and development, and how a healthy dietary pattern represented by MD and its components, especially olive oil, could exert a protective role by the development and progression of prostate cancer.

## Introduction

Prostate cancer is the second most common cancer in the world among men, and is the fifth most common cause of cancer death among men. Highest incidence rates are observed in Australia and New Zealand, Northern and Western Europe, and North America. The incidence rates of prostate cancer have increased in the last years mainly because of the practice of screening for prostate-specific antigen (PSA) in men without symptoms of the disease (1). The 5- and 10-year survival is higher in Europe and North America, and lower in some Asian and African countries (2). In Italy, prostate cancer is the most common cancer among men, with a prevalence rate of 1,200 per 100,000 persons and an estimated age-standardized incidence rate (on European population) of 89 per 100,000 person-years in 2015. Prostate cancer is the third cause of death for cancer among men in Italy; the estimated age-standardized mortality rate (on European population) is 14 per 100,000 person-years in 2015 (3). Almost all cases are adenocarcinomas, which originate in the peripheral zone of the prostate. It is considered that adenocarcinoma of the prostate derived mainly from the proliferation *in situ* and neoplastic degeneration of prostatic epithelial cells (4). Adenocarcinoma of the prostate metastasizes mainly to the lymph nodes and bones. Non-modifiable risk factors are age, race, and familial history. Genetic susceptibility of prostate cancer has been linked to African American. More than 30 single-nucleotide polymorphisms linked to prostate cancer susceptibility have been identified (5). In the US, African American men are 1.6 times more likely to develop prostate cancer than Caucasian men are. Many single-nucleotide polymorphisms that modestly affect risk have also been identified (6). Several epidemiological and clinical studies strongly support the association between nutritional factors and the development or progression of tumors, including breast cancer, prostate, and colorectal cancer (7). Also, many other tumor types have been recently included in a hypothetical list of diet-related cancers (8). Many components of the diet have been implicated to be protective or to promoting cancer development. Several pieces of evidence have shown that different food components, such as polyphenols, selenium, donors methyl-group, retinoids, the mono- and polyunsaturated fatty acids, isothiocyanates and allyl compounds, play a protective role toward the onset of cancer (9). It has been shown that these components can affect a variety of cellular processes, such as DNA repair, growth and cell differentiation, cell apoptosis, oxidative stress, inflammation, and so forth. However, in recent years, epigenetics has been indicated as the target of primary interest regarding the gene expression changes induced by the nutrients (10). The Mediterranean diet (MD) represents a dietary pattern suitable in the prevention of non-communicable diseases (11). A previous meta-analysis of observational studies (12), which investigated the effects of compliance to the MD on incidence and mortality of different types of cancer, showed that high adherence to MD was associated with a significant lower risk of overall cancer, especially colorectal cancer, pharyngeal and esophageal cancer, and prostate cancer. The aim of our narrative review was to analyze observational studies (cohort and case–control studies) that investigated the effects of overweight and obesity and diets components and the effect of adherence to MD on overall cancer risk, particularly on risk of prostate cancer.

## Body Fatness, Body Mass Index (BMI), and Prostate Cancer

### Obesity, Body Adiposity, and Prostate Cancer Development

The etiology of prostate cancer is still largely unknown (13). It seems to differ depending on the presentation of the disease at diagnosis, or if there is a localized carcinoma or an advanced prostate cancer (14). Several epidemiological and clinical studies have shown a link between obesity and the metabolic syndrome and risk of prostate cancer. It has been shown that obesity is associated with an increase in the incidence and mortality of prostate cancer (15). In addition, previous observational studies have reported associations between high BMI and high risk of cancer (16–18). From these and subsequent reports (19–21), two general links between BMI and cancer have been suggested: (1) increased insulin-like growth potentially stimulating cancer growth; (2) obesity is associated with a low-grade chronic inflammatory state, which in part is due to the infiltration of macrophages in adipose tissue. Obese individuals have elevated concentrations of circulating tumor necrosis factor-alpha, interleukin-6, and C-reactive protein, and leptine, compared with lean people, which are produced by the adipocytes. Accordingly, it is produced a state of low intensity chronic inflammation promoting the development of cancer. In men, obesity is linked to lower levels of serum testosterone. Since testosterone plays an important role in determining the differentiation of prostate epithelial cells, a decrease in testosterone levels can facilitate the growth of a form of less differentiated prostate cancer, which is of a more aggressive form of prostate cancer. A population-based prospective cohort study from a cohort of Swedish men aged 45–79 years (22) was conducted to examine the relationship between BMI at the age of 30 years and at the age from 45 to 79 years and the incidence of localized, advanced, and fatal prostate cancer.

Discacciati et al. (23). suggested a dual effect of body adiposity on prostate cancer development. Namely, a decreased risk for localized prostate cancer (RR: 0.94, 95% CI: 0.91–0.97, for every 5 kg/m^2^ increase) and an increased risk for advanced prostate cancer (RR: 1.09, 95% CI: 1.02–1.16, for every 5 kg/m^2^ increase). The biologic mechanisms behind the relationship between obesity and prostate cancer incidence remain unclear. Mendelian randomization is an epidemiological approach that aims to circumvent confounding by use of genetic variation in populations (24, 25). Davies et al. conducted a Mendelian randomization study based on 20,848 cases and 20,214 controls (26). They assumed that genetic variation in BMI could be used as exposure factor, which is not confounded by any environmental factor, for inquiring any causal association of obesity with prostate cancer risk. Namely, if BMI were a causal risk factor in the development of cancer, it would be expected that genetic variants that change BMI also might affect the risk of cancer. They found a weak and not statistically significant evidence that genetically elevated BMI was associated with a reduced risk of prostate cancer [odds ratio (OR) per SD increase in BMI genetic score: 0.98; 95% CI: 0.96–1.00; *p* = 0.07]. They also found that the genetically elevated BMI was associated with higher mortality for all causes among low-grade prostate cancer (OR per SD increase in the BMI genetic score: 1.08; 95% CI: 1.03–1.14, *p* = 0.002), but there were no associations with prostate cancer-specific mortality. This inverse relationship between BMI and prostate cancer risk was consistent with both observational data (27) and previous genetic studies (28). These genetic studies provide weak evidence that a higher BMI might be protective against the risk of prostate cancer or it could reduce the likelihood of the low-grade cancer for being detected; however, a higher BMI may increase the likelihood of death in men with low-grade prostate cancer. These observations support epidemiological findings that obesity protects against a diagnosis of localized prostate cancer but increases prostate cancer mortality (27). These findings were confirmed by a subsequent Mendelian randomization study (29), involving participants from two similar prospective studies of the Danish population, comprising 108,817 subjects. The authors found that high values BMI were not associated with a higher risk of prostate cancer in men (BMI 25–29.9: 1.06; 95% CI: 0.93–1.21; BMI ≥ 30: 0.95; 95% CI: 0.78–1.14; *p* trend 0.93), suggesting that previous observational associations could in some way be explained by confounding and behavioral factors.

### BMI and Localized Prostate Cancer

Concerning localized prostate cancer, authors observed a left-skewed “inverse U”-shaped relationship between BMI values and incidence of cancer at the age of 45–79 years. In correspondence with BMI value of 35 kg/m^2^, authors find a decreased incidence of 35% (RR: 0.65; 95% CI: 0.50–0.85) compared with the incidence at BMI value of 22 kg/m^2^, which was considered as reference value. In correspondence with BMI value of 18 kg/m^2^, authors find a decreased incidence of 23% (RR: 0.77; 95% CI: 0.54–1.11) compared with the reference value. They did not observe any statistically significant association between BMI at the age of 30 years and incidence of localized prostate cancer. As regards advanced prostate cancer, authors did not observe any statistically significant association between BMI at the age of 30 years, as well as at the age of 40–79 years, and incidence cancer.

### BMI and Fatal Prostate Cancer

Concerning fatal prostate cancer, a direct association between BMI and incidence cancer at the age of 45–79 years (BMI value 18 kg/m^2^ = RR: 0.89; 95% CI: 0.74–1.07; BMI value 35 kg/m^2^ = RR: 1.47; 95% CI: 0.81–2.69), and an inverse association at the age of 30 years (BMI value 18 kg/m^2^ = RR: 1.31; 95% CI: 1.02–1.70; BMI value 35 kg/m^2^ = RR: 0.41; 95% CI: 0.18–0.94), was observed. None of these results reached statistical significance. The results of this study could suggest a dual effect of obesity: an inverse relationship for BMI at the age of 30 years and a direct relationship for BMI during middle and late adulthood. These findings were confirmed from a successive dose–response meta-analysis on BMI and risk of prostate cancer (23).

## Red Meat, Processed Meat and Unprocessed Meat, and Prostate Cancer

The Diet and Cancer Report published by the World Cancer Research Fund and American Institute for Cancer Research (WCRF/AICR) in 2015 classified consumption of processed meat as “carcinogenic to humans.” For processed meat, we refer to meat that has been modified to improve the taste or the storage life, or both, through several processes as salting, curing, fermentation, and smoking (30). The Working Group classified consumption of red meat as “probably carcinogenic to humans.” For red meat, we refer to unprocessed mammalian muscle meat, which is derived from animals for slaughter, for example, beef, veal, pork, lamb, mutton, horse, or goat meat, or from hunting, for example, wild boar and deer. Red meat is usually consumed cooked. Both the consumption of red and processed is associated mainly with colorectal cancer, and with pancreatic and prostate cancer (30). Results from previous case–control and prospective cohort studies (31) have already suggest that a higher meat intake is associated to a greater risk of prostate cancer (RR = 1.2 or greater). Table 1 summarizes the further studies we examined.

**Table 1:** Red meat, processed meat and unprocessed meat intake, and development of cancer.

Reference	Characteristics of the studies	Sample size	Risk of prostate cancer
Kolonel (32)	Review from 14 case–control and 8 cohort studies	5,121 cases and 6,956 controls from case–control studies; 1,007 cases among 276,148 men from cohort studies	*Total prostate cancer*: RR = 1.3 or greater for higher vs lower quintiles of red meat intake

Nowell et al. (33)	Population-based case–control study	464 cases, 459 controls	*Total prostate cancer*: odds ratio (OR) = 1.68 (95% CI 1.20–2.36, *p* < 0.003) for well-done meat intake

Tavani et al. (34)	Review from case–control studies	127 cases, 3,220 male controls	*Total prostate cancer*: OR = 1.1 (95% CI 0.7–1.7, *p* = 0.24) for red meat intake

Bosetti et al. (35)	Case–control study	1,294 cases, 1,451 controls	*Total prostate cancer*: OR = 1.01 (95% CI 0.76–1.34, *p* = 0.79) for red meat intake

Cross et al. (36)	Prospective cohort study	29,361 men	*Total prostate cancer*: RR = 0.81 (95% CI 0.62–1.06, *p* = 0.38) for red meat intake RR = 1.69 (95% CI 1.19–2.40, *p* = 0.003) for very well-done meat intake

Sinha et al. (37)	Prospective cohort study	10,313 prostate cancer cases from a cohort of 175,343 men	*Total prostate cancer*: HR = 1.12 (95% CI: 1.04–1.21, *p* = 0.002) for red meat intake HR = 1.07 (95% CI: 1.00–1.14, *p* = 0.04) for processed meat intake *Advanced prostate cancer*: HR = 1.31 (95% CI: 1.05–1.65, *p* = 0.04) for red meat intake HR = 1.32 (95% CI: 1.08–1.61, *p* = 0.008) for processed meat intake HR = 1.24 (95% CI: 1.02–1.51, *p* = 0.03) for nitrite from meat intake HR = 1.31 (95% CI: 1.07–1.61, *p* = 0.03) for nitrate from meat intake *Total and advanced prostate cancer*: HR = 1.09 (95% CI: 1.02–1.17, *p* = 0.003) for barbecued meat intake HR = 1.28 (95% CI: 1.03–1.58, *p* = 0.02) for grilled meat intake

Wu et al. (38)	Pooled analysis of 15 cohorts from prospective studies of diet and cancer	52,683 prostate cancer cases from 842,149 subjects of 15 cohorts	RR = 1.02 (95% CI: 0.98–1.06, *p* = 0.93) for unprocessed red meat intake RR = 1.04 (95% CI: 1.01–1.08, *p* = 0.29) for processed red meat intake

Gilsing et al. (39)	Prospective cohort study	399 prostate cancer cases from a cohort of 120,852 subjects	*Advanced prostate cancer*: HR = 1.75 (95% CI: 1.03–2.97) for 1 day/week meat intake HR = 1.47 (95% CI: 0.35–3.30) for vegetarian HR = 1.77 (95% CI: 0.80–3.91) for fish intake

### Red Meat Intake and Development of Prostate Cancer

A review from 14 case–control and 8 cohort studies, concerning the relationship between meat intake and prostate cancer showed a risk ratio of 1.3 or greater for higher vs lower quintiles (32). Results from an interesting case–control study among US males have shown that high consumption of well-done meat was associated with an increased risk of prostate cancer (OR = 1.68, 95% CI 1.20–2.36; *p* < 0.003) (33). Authors have also shown that high consumption of well-done meat in subjects who are carriers of SULT1A1*1 genotype, which is associated with an high activity of Human sulfotransferase 1A1 (SULT1A1), that is involved in the activation of procarcinogens elements in some foods, have an higher risk of developing a prostate cancer (OR = 8.27, 95% CI 3.36–20.38; *p* = 0.02). Conversely, results from two studies study conducted among Italian males (34, 35), did not observe any significantly association between high intake of red meat and prostate cancer (OR = 1.1, 95% CI 0.7–1.7, *p* = 0.24; OR = 1.01, 95% CI 0.76–1.34, *p* = 0.79, respectively). The Prostate, Lung, Colorectal, and Ovarian Cancer Screening Trial (36), which examined a cohort of 29,361 US men, did not find any association between red meat intake and incidence of prostate cancer (RR = 0.81, 95% CI 0.62–1.06, *p* = 0.38); nevertheless, a significative association was shown between very well-done meat intake and incidence of prostate cancer (RR = 1.69, 95% CI 1.19–2.40, *p* = 0.003).

### Heterocyclic Amines (HCA), Polycyclic Aromatic Hydrocarbons (PAHs), and Development of Prostate Cancer

It has been suggested that the increased risk of colon cancer that is associated with high intake of red and processed meat could be related to HCA or to PAHs, which are generated during cooking at high temperatures or over an open flame, which had effects on hormone metabolism (40). Grilling or frying of meats could produce mutagenic compounds, such as HCA, or PAHs, which produced by pyrolysis of proteins and fats when grilling over coals; these compounds have been shown to cause DNA damage in prostate tissue culture (41). A prospective study (37) examined associations between meat consumption, considering type and cooking method, and related mutagens as heme iron and nitrite/nitrate, and prostate cancer risk, in a cohort of US men, as part of the NIH-AARP Diet and Health Study, during 9 years of follow-up. Authors found that high intake of red and processed meat was associated with an elevated risk of total prostate cancer (red meat: HR = 1.12, 95% CI: 1.04–1.21, *p* = 0.002; processed meat: HR = 1.07, 95% CI: 1.00–1.14, *p* = 0.04) and advanced prostate cancer (red meat: HR = 1.31, 95% CI: 1.05–1.65, *p* = 0.04; processed meat: HR = 1.32, 95% CI: 1.08–1.61, *p* = 0.008). Authors also found that heme iron, which is sourced from barbecued or grilled meat, was all significantly associated with a high risk of total and advanced prostate cancer (HR = 1.09, 95% CI: 1.02–1.17, *p* = 0.003; HR = 1.28, 95% CI: 1.03–1.58, *p* = 0.02, respectively).

### Nitrite and Nitrate and Development of Prostate Cancer

Nitrite and nitrate, which are used in meat processing, were also associated with high risk of advanced prostate cancer (HR = 1.24, 95% CI: 1.02–1.51, *p* = 0.03; HR = 1.31, 95% CI: 1.07–1.61, *p* = 0.03, respectively). On the other hand, results from a pooled analysis of 15 prospective cohort study (38) involving 842,149 men from North America, Europe, Australia, and Asia, examined the association of incidence of prostate cancer and the intake of unprocessed and processed red meat, seafood, eggs, and poultry. Authors did not find a significative association among unprocessed red meat and processed red meat intake and prostate cancer risk (≥120 vs <20 g/day: RR = 1.02, 95% CI: 0.98–1.06, *p* = 0.93; RR = 1.04, 95% CI: 1.01–1.08, *p* = 0.29, respectively). About poultry and seafood, it was not observed any association with prostate cancer risk (≥45 vs <5 g/day: RR = 1.05, 95% CI: 1.00–1.09, *p* = 0.33; ≥40 vs <5 g/day: RR = 1.04, 95% CI: 0.98–1.09, *p* = 0.67, respectively). A positive association was detected between eggs intake and fatal prostate cancers risk (≥25 vs <5 g/day: RR = 1.14, 95% CI: 1.00–1.03, *p* = 0.01). On the other hand (39), a population-based cohort study of 11.082 subjects, did not observe any statistically significant reduction of risk of advanced prostate cancer among low week meat consumers compared with individuals with the highest meat intake. Paradoxically, it was found that low meat consumers (1 day/week), vegetarians, and fish consumers had an increased risk of advanced prostate cancer (HR: 1.75, CI: 1.03–2.97; HR: 1.47, CI 0.35–3.30; HR: 1.77, CI: 0.80–3.91, respectively) than subjects with the highest meat intake (6/7 days/week), also after adjustment for confounders (age, total energy intake, cigarette smoking, alcohol consumption, BMI, non-occupational physical activity, level of education, family history of prostate cancer). It was also found that prostate cancer rick was further increased after adjustment for dietary factors, including milk, cheese, and eggs intake, and for lifestyle factors, including cigarette smoking, among vegetarians (HR = 2.44), fish consumers (HR = 2.90), and 1 day/week consumers (HR = 2.43). The authors explain this paradoxical result by suggesting that vegetarians are also less likely to follow prostate cancer screening guidelines compared with non-vegetarians.

## Fatty Acids, Dairy Products, and Calcium

### Fatty Acids Intake and Risk of Prostate Cancer

As summarized in Table 2, a prospective study (42) conducted on 47,855 men within the Health Professional Follow-up Study, proved that high intake of α-linolenic was strongly related to risk of advanced prostate cancer (high quintile vs low quintile: RR = 3.43, 95% CI = 1.67–7.04, *p* = 0.002). Authors also find a positive association between fat intake from red meat and an elevated risk of advanced prostate cancer (high quintile vs low quintile of red meat fat intake: RR = 2.64, 95% CI = 1.21–5.77, *p* = 0.02). Authors also found an inverse but not statistically significant association between the intake of omega-3 fatty acids from fish [eicosapentaenoic acid (EPA) and docosahexaenoic acid (DHA)] and the risk of advanced prostate cancer (high quintile vs low quintile: RR = 0.90, 95% CI = 0.51–1.61, *p* = 0.3). The analysis of the plasma fatty acids from the data of the Physician’s Health Study (43) has confirmed that high intake of α-linolenic acid from meat and dairy food was positively associated with the risk of prostate cancer. It was reported that RR for highest quartiles vs lowest quartiles of α-linolenic acid levels was 2.1 (95% CI = 0.9–4.9), compared with those with levels below the detection threshold (*p* trend = 0.03). The OR for consuming beef, pork, or lamb (red meat) at least five to six times per week compared with one to three times per month or less was 2.51 (95% CI = 0.93–6.74). Particularly, Gann et al. confirmed that intake of high intake of α-linolenic acid and meat was strongly related to risk of prostate cancer (high quartile vs low quartile: RR = 2.22, 95% CI = 0.93–5.29, *p* = 0.04). In addition, omega-3 fatty acids from fish (EPA) was inversely but not significantly associated with a risk of advanced prostate cancer (high quartile vs low quartile: RR = 0.87, 95% CI = 0.41–1.82, *p* = 0.81). Concerning the relationship of α-linolenic acid and the risk of prostate cancer, a systematic review and subsequent meta-analysis involving 16 studies (44) revealed that highest concentrations of α-linolenic acid were associated with an increased risk of prostate cancer (RR = 1.20; 95% CI: 1.01, 1.43; *p* = 0.04). However, after adjustment for publication bias, this association was no longer was evident (RR = 0.94; 95% CI: 0.79, 1.17; *p* = 0.68).

**Table 2:** Fatty acids intake and development of prostate cancer.

Reference	Characteristics of the studies	Sample size	Risk of prostate cancer
Giovannucci et al. (42)	Prospective cohort study	126 cases from a cohort of 47,855 subjects	*Advanced prostate cancer*: RR = 0.95 (95% CI = 0.41–2.21, *p* = 0.56) for high intake of saturated fats RR = 3.43 (95% CI = 1.67–7.04, *p* = 0.002) for high intake of α-linolenic acid RR = 2.64 (95% CI = 1.21–5.77, *p* = 0.02) for high intake of fats from red meat RR = 0.90 (95% CI = 0.51–1.61, *p* = 0.3) for high intake of eicosapentaenoic acid and docosahexaenoic acid from fish

Gann et al. (43)	Prospective cohort study	120 prostate cancer cases and 120 controls from a cohort of 14,916 subjects	*Total prostate cancer*: RR = 2.1 (95% CI = 0.9–4.9, *p* = 0.03), for highest quartiles of α-linolenic acid intake RR = 2.22 (95% CI = 0.93–5.29, *p* = 0.04) for high intake of α-linolenic acid and meat RR = 0.87 (95% CI = 0.41–1.82, *p* = 0.81) for high intake of eicosapentaenoic acid

Simon et al. (44)	Meta-analysis from 13 retrospective case–control studies and 3 prospective cohort studies	5,701 prostate cancer cases and 7,449 controls from case–control studies 9,267 prostate cancer cases among 159,941 subjects from prospective cohort studies	*Total prostate cancer*: data from overall analysis; RR = 1.20 (95% CI = 1.01–1.43, *p* = 0.04) for highest quantiles of α-linolenic acid intake *Total prostate cancer: data after adjustment for publication bias;* RR = 0.94 (95% CI = 0.79–1.17, *p* = 0.68) for highest quantiles of α-linolenic acid intake

Szymanski et al. (45)	Meta-analysis from 12 case–control studies and from 12 cohort studies	5,777 cases of prostate cancer cases and 9,805 controls from case–control studies 13,924 prostate cancers cases from a cohort of 445,820 men	*Total prostate cancer: data from case–control studies* Odds ratio = 0.85 (95% CI = 0.72–1.00, *p* = 0.05) for high consumption of fish *Total prostate cancer: data from cohort studies* RR = 1.01 (95% CI = 0.90–1.14, *p* = 0.83) for high consumption of fish *Prostate cancer mortality: data from pooled results of four cohort studies* RR = 0.37 (95% CI = 0.18–0.74, *p* = 0.005) for high consumption of fish

Kurahashi et al. (46)	Population-based prospective study	329 cases from a cohort of 43,435 men	*Total prostate cancer*: RR = 1.62 (95% CI = 1.15–2.29, *p* < 0.01) for high intake of myristic acid RR = 1.53 (95% CI = 1.07–2.20, *p* = 0.04) for high intake of palmitic acid

Regarding the relationship between saturated fatty acids (SFA) and the risk of prostate cancer, a population-based prospective study among 43,435 Japanese men (46) was conducted to investigate if the intake of specific SFA could increase in a dose-dependently way the risk of prostate cancer. Authors showed that high intake of myristic acid (which is found in dairy products, especially butter, cream and cheese, coconut oil, and palm kernel oil) and palmitic acid (which is found in palm oil, but it is also contained in butter, cheese, milk, and meat), was associated with an augmented risk of prostate cancer (RR = 1.62, 95% CI: 1.15–2.29, *p* < 0.01; RR = 1.53, 95% CI: 1.07–2.20, *p* = 0.04).

A meta-analysis from 12 case–control studies (5,777 cases and 9,805 control) and from 12 cohort studies (445,820 subjects), concerning fish intake and the incidence and mortality of prostate cancer (45), did not observe any significant association between fish consumption and a reduction of prostate cancer incidence among cohort studies (RR: 1.01; 95% CI: 0.90–1.14; *p* = 0.83). Authors observed a weak association between fish intake and reduction of prostate cancer incidence from case–control studies (RR: 0.85; 95% CI: 0.72–1.00; *p* = 0.05). Also, authors observed a significant reduction of mortality from prostate cancer associated with a high consumption of fish, by pooling the four of the cohort studies (RR: 0.37; 95% CI: 0.18, 0.74; *p* = 0.005).

### Dairy Products, Calcium and Vitamin D Intake and Risk of Prostate Cancer

Regarding the intake of dairy products and prostate cancer risk, within the study mentioned above conducted by Kurahashi et al., a strong positive association between high intake of dairy products and prostate cancer was observed (339.8 vs 12.8 g/day: RR = 1.63, 95% CI = 1.14–2.32, *p* = 0.01). In addition, authors observed a strong positive association between high intake of milk (290.5 vs 2.3 g/day: RR = 1.53, 95% CI = 1.07–2.19, *p* = 0.001) and between high intake of yogurt (31.5 vs 1.9 g/day: RR = 1.52, 95% CI = 1.10–2.12, *p* < 0.001) and risk of prostate cancer. Intake of cheese was not statistically associated with total prostate cancer (6.2 vs 1.9 g/day: RR = 1.32, 95% CI = 0.93–1.89, *p* = 0.30), nor the calcium intake (725.1 vs 282.8 mg/day: RR = 1.24, 95% CI = 0.85–1.81, *p* = 0.16).

Subsequently (47), a cohort study was conducted among 2,806 subjects with prostate cancer, from the Physicians’ Health Study, to investigate the relation between intakes of several types of dairy products and the incidence and survival of prostate cancer during a 28-years follow-up. They found that total dairy food intake was marginally and not statistically associated with prostate cancer risk (highest vs lowest intake: HR = 1.12, 95% CI: 0.93–1.35, *p* = 0.06). In addition, whole milk intake was not associated with prostate cancer risk (highest vs lowest intake: HR = 0.95, 95% CI: 0.81–1.10, *p* = 0.32), nor calcium from dairy food intake (highest vs lowest intake: HR = 1.14, 95% CI: 0.97–1.34, *p* = 0.07). Authors found that higher intake of skim/low-fat milk was associated with a higher risk of prostate cancer (highest vs lowest intake: HR = 1.19, 95% CI: 1.06–1.33, *p* = 0.001). In particular, high consumption of skim/low-fat milk was associated with a higher risk of low-grade and localized prostate cancer (highest vs lowest intake: HR = 1.20, 95% CI: 1.06–1.37, *p* = 0.001; HR = 1.19, 95% CI: 1.04–1.35, *p* = 0.004, respectively). By contrast, for risk of fatal prostate cancer, whole milk intake was strongly and statistically associated with high mortality for prostate cancer (highest vs lowest intake: HR = 2.17, 95% CI: 1.34–3.51, *p* < 0.001). A wider review conducted later (48) asserted the association between a high intake of dairy foods and prostate cancer risk, as shown in the NIH-AARP cohort (highest vs lowest intake: HR = 1.06, 95% CI: 1.01–1.12, *p* = 0.01) and the lack of association between calcium from food intake and the risk of prostate cancer (RR = 1.04; 95% CI: 0.98–1.09, *p* = 0.14) (49). In addition, Abid et al. confirmed the lack of association between milk intake and the risk of prostate cancer (RR = 1.06, 95% CI: 0.91–1.23, *p* = ns), as well the lack of association between dairy products and calcium intake and the risk of prostate cancer (RR = 1.06, 95% CI = 0.92–1.22, *p* = ns; RR = 1.04, 95% CI = 0.90–1.15, *p* = ns, respectively), as previously showed (50).

An experimental study conducted on transgenic mice expressing prostate adenocarcinoma at intraepithelial stage, which were fed with high amount of milk (skim or whole) for 15–27 weeks (51), showed that high milk consumption, either skim or whole, did not aggravate nor promote tumor progression. Even, milk intake could exhibit slight protective effects by not promoting the expression of tumor-related markers like Ki-67 and Gprc6a. Authors concluded asserting that regular milk consumption should be not detrimental for patients with early-stage prostate tumors.

Then, a meta-analysis from 32 prospective studies was conducted within the Continuous Update Project (52) to investigate the relation between dairy, calcium intakes, and prostate cancer risk, and to investigate any association among the types of dairy products and the sources of calcium intake with the prostate cancer risk. They showed that high intake of total dairy products (summary RR = 1.07, 95% CI: 1.02–1.12, per 400 g/day), total milk (summary RR = 1.03, 95% CI: 1.00–1.07, per 200 g/day), low-fat milk (summary RR = 1.06, 95% CI: 1.01–1.11, per 200 g/day), cheese (summary RR = 1.09, 95% CI: 1.02–1.18, per 50 g/day), and dietary calcium (summary RR = 1.05, 95% CI: 1.02–1.09, per 400 mg/day) were associated with increased total prostate cancer risk. They also showed that high intake of calcium from dairy products, but not not-dairy calcium or supplemental calcium intakes, were associated with total prostate cancer risk.

Regarding calcium intake and the risk of prostate cancer, it has been proposed that high calcium intake down regulates the formation of 1,25-dihydroxyvitamin D2 (e brgocaliferol D2), which is the active form of vitamin D. Ergocalciderol-D2 could play an important role in prostate cancer carcinogenesis by inhibiting cell proliferation. Down regulation of the ergocaliferol-D2, thereby could increase the cell proliferation in the prostate cancer (53).

The meta-analysis conducted by Huncharek et al. (50) had not revealed any significant relationship between vitamin D intake and prostate cancer (RR = 1.16, 95% CI = 0.98–1.28, *p* = 0.37).

A subsequent meta-analysis of 21 studies (54) reported a statistically association between higher Vitamin D concentrations and a higher risk for developing prostate cancer (OR: 1.17, 95% CI: 1.05–1.30, *p* = 0.004). Taken together, epidemiological studies did not provide any strong evidence that higher concentrations of Vitamin D might reduce the risk of prostate cancer.

At present, there is no evidence that vitamin D will reduce the incidence of prostate cancer, and there is an inconsistent evidence that Vitamin D may prevent progression of early-stage disease and mortality (55). All the studies above mentioned are summarized in Table 3.

**Table 3:** Dairy products, calcium intake, and Vitamin D and development of prostate cancer.

Reference	Characteristics of the studies	Sample size	Risk of prostate cancer
Kurahashi et al. (46)	Population-based prospective study	329 cases from a cohort of 43,435 men	*Total prostate cancer*: RR = 1.63 (95% CI = 1.14–2.32, *p* = 0.01) for high intake of dairy products RR = 1.53 (95% CI = 1.07–2.19, *p* = 0.001) for high intake of milk RR = 1.52 (95% CI = 1.10–2.12, *p* < 0.001) for high intake of yogurt RR = 1.32 (95% CI = 0.93–1.89, *p* = 0.30) for high intake of cheese RR = 1.24 (95% CI = 0.85–1.81, *p* = 0.16) for high intake of calcium

Song et al. (47)	Prospective cohort study	Survival analysis among 2,806 incident prostate cancer cases, from a cohort of 21,660 men	*Total prostate cancer*: HR = 1.12 (95% CI = 0.93–1.35, *p* = 0.06) for high intake of dairy products HR = 0.95 (95% CI = 0.81–1.10, *p* = 0.32) for high intake of whole milk HR = 1.14 (95% CI = 0.97–1.34, *p* = 0.07), for high intake of calcium from dairy products HR = 1.19 (95% CI = 1.06–1.33, *p* = 0.001) for high intake of skim/low-fat milk *Low-grade prostate cancer*: HR = 1.20 (95% CI = 1.06–1.37, *p* = 0.001) for high skim/low-fat milk intake *Localized prostate cancer*: HR = 1.19 (95% CI = 1.04–1.35, *p* = 0.004) for high skim/low-fat milk intake *Fatal prostate cancer*: HR = 2.17 (95% CI = 1.34–3.51, *p* < 0.001) for high whole milk intake

Park et al. (49)	Prospective cohort study	17,189 cases in a total cohort of 293,907 men and 198,903 women	*Total prostate cancer*: RR = 1.06 (95% CI = 1.01–1.12, *p* = 0.01) for high intake of dairy products RR = 1.03 (95% CI = 0.98–1.08, *p* = 0.21) for high intake of calcium

Huncharek et al. (50)	Meta-analysis from 45 observational studies	26,769 cases from 21 cohort studies and from 24 case–control studies	*Total prostate cancer*: RR = 1.06 (95% CI = 0.92–1.22, *p* = ns) for high intake of dairy products RR = 1.06 (95% CI = 0.91–1.23, *p* = ns) for high intake of milk RR = 1.04 (95% CI = 0.90–1.15, *p* = ns) for high intake of calcium RR = 1.16 (95% CI = 0.98–1.28, *p* = 0.37) for intake of Vitamin D

Aune et al. (52)	Meta-analysis from 32 prospective studies within the continuous update project	63,308 prostate cancer cases among 2,338,285 subjects	*Total prostate cancer*: summary RR = 1.07 (95% CI = 1.02–1.12) for 400 g/day intake of dairy products RR = 1.03 (95% CI = 1.00–1.07) for 200 g/day intake of milk Summary RR = 1.06 (95% CI = 1.01–1.11) for 200 g/day intake of low-fat milk Summary RR = 1.09 (95% CI = 1.02–1.18) for 50 g/day intake of cheese Summary RR = 1.05 (95% CI = 1.02–1.09) for 400 g/day intake of dietary calcium Summary RR = 1.06 (95% CI = 1.02–1.09) for 400 g/day intake of dairy calcium Summary RR = 0.97 (95% CI = 0.90–1.04) for 400 g/day intake of non-dairy calcium Summary RR = 0.99 (95% CI = 0.96–1.01) for 400 g/day intake of supplemental calcium

Rodriguez et al. (53)	Prospective cohort study	3,811 cases from a cohort of 65,321 men	*Total prostate cancer*: RR = 1.2 (95% CI = 1.0–1.6, *p* = 0.02) for ≥2,000 mg/day intake of calcium RR = 1.5 (95% CI = 1.1–2.0, *p* < 0.01) for ≥2,000 mg/day intake of calcium for men not having prostate-specific antigen testing before 1992 RR = 1.6 (95% CI = 1.1–2.3, *p* = 0.10) for ≥2,000 mg/day intake of dietary calcium RR = 1.1 (95% CI = 0.9–1.3, *p* = 0.38) for 4+ servings/day of dairy intake *Advanced prostate cancer*: RR = 1.6 (95% CI = 0.9–3.0, p = 0.08) for ≥2,000 mg/day intake of calcium RR = 2.2 (95% CI = 0.9–5.3, *p* = 0.27) for ≥2,000 mg/day intake of dietary calcium RR = 0.9 (95% CI = 0.5–1.4, *p* = 0.95) for 4+ servings/day of dairy intake

Xu et al. (54)	Meta-analysis from case–control studies and prospective cohort studies	11,941 cases and 13,870 controls	*Total prostate cancer*: odds ratio (OR) = 1.17 (95% CI = 1.05–1.30, p = 0.004) for overall studies OR = 1.17 (95% CI = 1.08–1.27, *p* < 0.001) for nested case–control studies OR = 1.22 (95% CI = 0.96–1.55, *p* = 0.097) for cohort studies OR = 1.15 (95% CI = 1.03–1.29, *p* = 0.017) for USA studies OR = 1.21 (95% CI = 1.04–1.40, *p* = 0.014) for Europe studies OR = 1.20 (95% CI = 1.01–1.42, *p* = 0.042) for serum-sample studies OR = 1.13 (95% CI = 1.00–1.27, *p* = 0.05) for plasma-sample studies

## Mediterranean Diet

**Figure 1:**
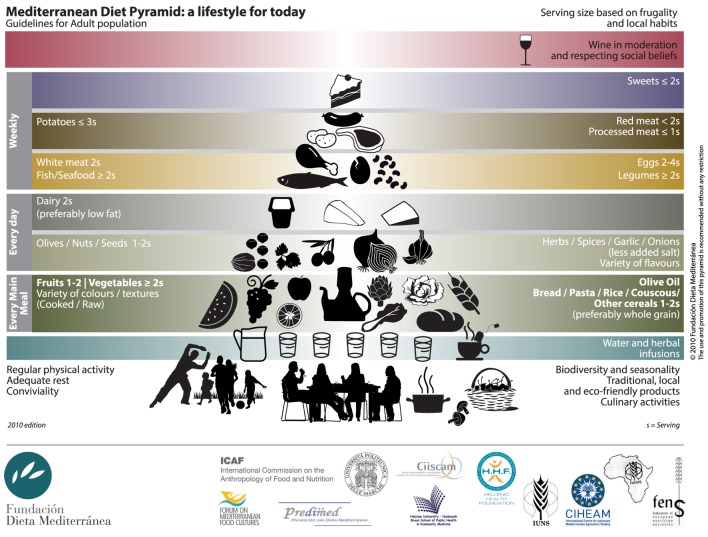
Mediterranean diet pyramid.

The traditional MD is characterized by a high intake of foods of plant origin (fruit, vegetables, breads, other cereals, potatoes, beans, nuts, and seeds) and fresh fruit as daily dessert. Olive oil is the principal source of fats. Dairy products (mainly light cheese and yogurt), fish and poultry are consumed in low-to-moderate amounts, egg consumption is limited to a maximum of four per week, red meat is consumed in low amounts, sporadically, or no more than once a week. MD is low in saturated fats, which are no more than 8% of the total caloric intake. The caloric intake derived from fats does not exceed 30% of the total caloric intake (56). Wine is consumed in low-to-moderate amounts, normally with meals [(57), Figure 1]. Epidemiological studies (58) have suggested beneficial health effects derived from the MD.

### MD and BMI

From Ancel Keys’ studies, the MD has been proposed as a healthy diet model; has been associated with a lower risk of cardiovascular and metabolic diseases. The traditional MD has also been proposed as an optimal weight-loss diet model. An interesting population-based cross-sectional survey conducted in a Spanish population to assess the relation between BMI and obesity and adherence to the traditional MD, by a multiple linear regression analysis (59). 1,547 men and 1,615 women, aged 25–74 years, were examined. After controlling for potential confounders, authors showed that a high adherence to the traditional MD pattern was associated with a change in the BMI of 0.43 in men (β coefficient: −0.043, SD: 0.040, *p* = 0.030, *R*^2^ for model: 0.082) and 0.68 for women (β coefficient: −0.068, SD: 0.050, *p* = 0.007, *R*^2^ for model: 0.171). In addition, a high adherence to the MD, assessed by the score quartiles of the Mediterranean Diet Score (MDS), was associated with a lower prevalence of obesity both in men and in women. On the other hand, a prospective study conducted on a Greek population sample of 9,612 men and 13,985 women (60) did not show significant association (*p* = 0.09) between MD adherence, measured by the MDS, and the BMI among either men or women, after correction for confounders, as sex, age, years of schooling, smoking, and physical activity. Author concluded that overweight in the Greek population could be related exclusively to limited physical activity and to excessive energy intake.

Similarly, a prospective study conducted on a Spanish population, involving 6,319 subjects (61), did not find a significant association among adherence to a MD pattern, weight gain, and BMI, after correction for baseline age, gender, BMI, smoking, physical activity, alcohol and energy intake, and changes in dietary habits. Also, Spanish authors concluded that obesity might be related to declining physical activity to the “westernization” of the traditional MD, namely the reduction in the intake of fruit and vegetables and increase in consumption of sugar, animal proteins, and saturated fats. Data from Greek and Spanish population were confirmed in a sample of 3,090 men and 3,529 women, obtained from the control group of a network of case–control studies conducted in northern, central, and southern Italy (62). Authors confirmed that adherence to a traditional MD pattern is unrelated to BMI, after adjusting data for age, study center, education, tobacco smoking, occupational physical activity, and total energy intake. On the other hand, results from a study conducted on an Israeli group of 322 subjects (63) to compare the effectiveness and the safety of three models of diet (MD, low-fat diet, and low-carbohydrate diet.) showed that, after 36 months, MD and low-carbohydrate diet were more effective for weight loss (*p* < 0.001). MD was more effective, after 24 months for reducing fasting glucose levels (*p* < 0.001) and insulin resistance, measured by the HOMA-IR (*p* = 0.04) among the diabetic subjects. These data were subsequently confirmed by a systematic review of 20 randomized controlled trials (64), involving 3,073 subjects with type 2 diabetes. Authors showed that MD had the largest effect for improving the in glycemic control, evaluated by the glycated hemoglobin reduction (HbA1c) (*p* < 0.00001). Also, MD induced a greater weight loss (*p* < 0.00001). A partial further confirmation has been given by a meta-analysis of nine randomized controlled trials, involving 1,178 patients, to explore the effects of MD on glycemic control, weight loss, and cardiovascular risk factors in subjects with type 2 diabetes (65). Results from meta-analysis showed that MD had the greater effects in improving HbA1c (*p* = 0.001) and fasting glucose levels (*p* = 0.007). Authors also stated that MD was effective to reduce BMI (*p* = 0.976) and body weight (*p* = 0.924). Lastly, an interesting study involving 77 men and 114 women within the PREDIMED study (66) in order to compare the effects of dietary interventions by MD supplemented with virgin olive oil, MD supplemented with nuts and a low-fat diet, revealed that all the three diets induced a reduction of waist circumference (*p* = 0.003, *p* = 0.001 and *p* = 0.001 for low-fat, olive oil and nut diets, respectively), but only in the MD groups, was observed a significant reduction in body weight (*p* = 0.003 and *p* = 0.021 for olive oil and nut diets, respectively).

### MD Adherence and Cancer

A large, population-based prospective study (67) showed that a high degree of adherence to the traditional MD, which was quantified by the MD Scale (68), was associated with a reduction in total mortality. Specifically, authors found that a two-point increment in the Mediterranean-diet score was associated with a 25% reduction in total mortality (HR 0.75; 95% CI 0.64–0.87; *p* < 0.001). This inverse association between the Mediterranean-diet score and total mortality was evident also after adjustment for confounding factors as sex, smoking status, level of education, body mass index, waist-to-hip ratio, and level of physical activity. Particularly, a reduction of mortality for cancer was observed. Specifically, a two-point increment in the Mediterranean-diet score corresponded to 24% reduction in mortality for cancer (HR 0.76; 95% CI 0.59–0.98). In 2004 results from a case–control involving 1,294 patients with histologically confirmed carcinoma of the prostate and 1,451 controls (35), showed a significant increased risk for more frequent consumption of milk and dairy products (OR: 1.15 for highest vs lowest quintile, *p* = 0.03) as well as bread (OR: 1.38, *p* = 0.01). Also, an inverse association for soups (OR: 0.77, *p* = 0.02) and cooked vegetables (OR: 0.74, *p* = 0.01) was observed. In addition, in 2015 a meta-analysis was conducted to review the relationship between consumption of vegetables and fruit and the risk of several cancers, in a network of Italian and Swiss case–control studies including over 10,000 cases and about 17,000 controls (69).

Authors found a significant reduction of risk for prostate cancer comparing regular (≥1 portion/week) vs occasional intake (<1 portion/week) of Cruciferous vegetables (OR: 0.87, 95% CI: 0.70–1.09) and comparing regular vs occasional intake of Onions (≥7 portion/week vs no use; OR: 0.29, 95% CI: 0.07–1.03) and Garlic (high vs no or low use; OR: 0.81, 95% CI: 0.64–1.00).

As regard fruit intake, authors showed that apple intake was associated with a reduced risk of prostate cancer (OR: 0.91; 95% CI: 0.77–1.07). Authors also found a significant inverse association between high intake of proanthocyanidins, which are found in apples, red wine, cranberry, black currant, green tea, black tea, and prostate cancer (OR: 0.87; 95% CI 0.76–0.99). Apples contain high levels of flavonoids and phenolic acids and they had a high level of antioxidant activity. Authors concluded by assuming that the protective effects of vegetables and fruit against cancer are correlated with the whole of their constituents. Cruciferous vegetables, such as cabbages, cauliflowers, broccoli, Brussels sprouts, and turnip greens, are important sources of isothiocyanates, which have anti-carcinogenic properties. Garlic and onion are a source of several organosulphur compounds and have anti-inflammatory, anti-thrombotic, cholesterol-lowering, and antioxidant properties.

Schwingshackl and Hoffmann in their meta-analysis (12) have confirmed the concept that a high adherence to the MD diet was associated with a significant reduction in both mortality and cancer incidence by 10% (RR: 0.90, 95% CI 0.86–0.95, *p* < 0.0001). Their results were in accordance with previous meta-analyses, which analyzed the effects of high adherence to MD on reduction of cancer risk (70, 71). In particular, Schwingshackl and Hoffmann assessed that the risk of prostate cancer could be reduced by 4% with a high adherence to MD. In their review, the authors confirmed both the concept that dietary factors could reduce cancer risk through several mechanisms, involving the suppression of spontaneous mutations of DNA, the modulation of cell proliferation, or the methylation of DNA and the induction of apoptosis. Schwingshackl and Hoffmann also reiterated the highly protective role of the olive oil, which is one of the main components of the MD.

### Olive Oil Consumption and Cancer Mortality

A systematic review and meta-analysis involving 13,800 patients and 23,340 controls from 19 observational studies (72) suggested a lower likelihood of having any type of cancer after comparing the highest category of olive oil consumption with the lowest (log OR = −0.41; 95% CI −0.53, −0.29). They also reported the results from a case–control study, involving 320 Greek patients with histologically confirmed incident prostate cancer and 246 controls (73). After adjustment for total energy intake, authors find that olive oil was unrelated to the risk (*p* = 0.66); the specific cancer-protective effect could be attributed to the high content of vitamin E in olive oil, which was significantly inversely related to prostate cancer risk (OR 0.53, 95% CI 0.30 to 0.94, *p* = 0.03). On the other hand, Psaltopoulou et al. have confirmed that an increased consumption of monounsaturated fatty acids (MUFA)-rich vegetables oils, but not MUFA of animal origin, had a protective role toward prostate cancer risk.

Already in 2008 (74) was pointed out the role of a plethora of minor constituents in olive oil in addition to oleic acid, as protective agents against initiation, promotion, and progression of the carcinogenic process.

These components include alpha-tocopherol, and carotenoids, which have been widely studied, and several phenolic compounds, such as tyrosol and hydroxytyrosol, which represent the major share of antioxidants in olive oil as metabolites of the oleuropein, phytosqualene, secoiridoids, phytosterols, and lignans. A pooled analysis conducted from 15 studies, involving 11.239 case and 18.541 controls (75), showed that alpha-tocopherol was associated with a reduced risk of prostate cancer (highest vs lowest quintiles: OR = 0.86, 95% CI = 0.78–0.94, *p* < 0.001). The authors have shown that alpha-tocopherol is particularly protective against both advanced and aggressive prostate cancer (80% increase of alpha-tocopherol: OR = 0.71, 95% CI = 0.57–0.88, *p* = 0.019; OR = 0.70, 95% CI = 0.58–0.86, *p* = 0.014, respectively).

### Lycopene and Prostate Cancer

**Figure 2:**
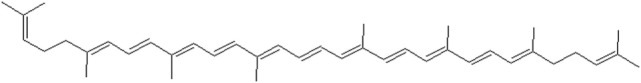
Lycopene.

Lycopene (Figure 2) is a tetra-terpene from the carotenoid family, which is found in tomatoes and in red fruits and vegetables, such as red carrots, watermelons, strawberries, cherries, pomegranates, blood oranges, and papayas, is responsible for reducing the risk of various cancers, particularly prostate cancer.

Intestinal absorption and, hence, the bioavailability of lycopene is improved by fats and by cooking of foods that contain it, for example, by cooking the tomato sauce. It was observed that the plasma concentration of lycopene significantly increased after the consumption of tomatoes meals cooked in olive oil, compared to the consumption of tomatoes meals cooked without olive oil (76).

A case–control study conducted within the Physicians’ Health Study (77) had already shown that the risk for aggressive prostate cancers was significantly reduced in subjects with high concentration of lycopene (fifth quintile vs first quintile: Unadjusted OR = 0.56, 95% CI = 0.34–0.92, *p* = 0.02). Particularly, it has been shown a significant association between high plasma levels of lycopene and a strong reduction of aggressive prostate cancers (fifth quintile vs first quintile: OR = 0.40, 95% CI = 0.19–0.84, *p* = 0.006). Afterward, Key et al. (75) confirmed that lycopene exerts a protective role against advanced and aggressive prostate cancer (80% increase of lycopene: OR = 0.73, 95% CI = 0.54–0.99, *p* = 0.036; OR = 0.72, 95% CI = 0.53–0.97, *p* = 0.025, respectively). All the abovementioned pieces of evidence are summarized in Table 4.

**Table 4:** Mediterranean diet (MD) components and development of prostate cancer.

Reference	Characteristics of the studies	Sample size	Risk of prostate cancer
Bosetti et al. (35)	Case–control study	1,294 cases and 1,451 controls	Odds ratio (OR) = 1.15 (95% CI = 0.90–1.46, *p* = 0.03) for highest vs lowest quintiles of milk or dairy products intake OR = 1.38 (95% CI = 1.03–1.83, *p* = 0.01) for highest vs lowest quintiles of bread intake OR = 0.77 (95% CI = 0.59–0.99, *p* = 0.02) for highest vs lowest quintiles of soups intake OR = 0.74 (95% CI = 0.57–0.95, *p* = 0.01) for highest vs lowest quintiles of cooked vegetables intake

Turati et al. (69)	Meta-analysis from case–control studies	1.294 prostate cancer cases from 10,796 cases of different cancers and 17,000 controls	OR = 0.87 (95% CI = 0.70–1.09) for highest intake of cruciferous vegetables OR = 0.29 (95% CI = 0.07–1.03) for highest intake of onions OR = 0.81 (95% CI = 0.64–1.00) for highest intake of garlic OR = 0.91 (95% CI = 0.77–1.07) for highest intake of apples OR = 0.87 (95% CI = 0.76–0.99) for highest intake of proanthocyanidins

Schwingshackl and Hoffmann (12)	Meta-analysis from 21 cohort studies and 12 case–control studies	29,867 prostate cancer cases from a total number of 1,431,461 subjects	RR: 0.96 (95% CI = 0.92–0.99, *p* = 0.03) for high adherence to the MD

Psaltopoulou et al. (72)	Systematic review and meta-analysis from 19 observational studies	13,800 patients and 23,340 controls from overall studies; 1,495 prostate cancer cases and 1,631 controls from three case–control studies	OR: 0.53 (95% CI = 0.30–0.94, *p* = 0.03) for high content of vitamin E in olive oil

Key et al. (75)	Meta-analysis from 15 case–control studies	11,239 prostate cancer cases and 18,541 controls	*Total prostate cancer*: OR = 1.13 (95% CI = 1.04–1.22, p = 0.015) for highest vs lowest quintiles of retinol intake OR = 0.86 (95% CI = 0.78–0.94, *p* < 0.001) for highest vs lowest quintiles of α-tocopherol intake *Advanced prostate cancer*: OR = 0.71 (95% CI = 0.57–0.88, p = 0.019) for 80% increase of α-tocopherol OR = 0.73 (95% CI = 0.54–0.99, *p* = 0.036) for 80% increase of lycopene *Aggressive prostate cancer*: OR = 0.70 (95% CI = 0.58–0.86, p = 0.014) for 80% increase of α-tocopherol OR = 0.72 (95% CI = 0.53–0.97, *p* = 0.025) for 80% increase of lycopene

Gann et al. (77)	Case–control study	578 prostate cancer cases and 1,294 controls	*Aggressive prostate cancer*: unadjusted OR = 0.56 (95% CI = 0.34–0.92, p = 0.02) for highest concentration of lycopene OR = 0.40 (95% CI = 0.19–0.84, *p* = 0.006) for highest vs lowest plasma levels of lycopene

It is, therefore, evident that, apart from being stabilizers of oleic acid by protecting the unsaturated fats against oxidants, phenolic compounds present in extra virgin olive oil may exert a strong chemo-preventive effect *via* a variety of distinct mechanism as well, including both direct antioxidant effects and actions on cancer cell signaling and cell cycle progression.

### MD, Inflammation, and DNA Damage in Prostate Cancer

An interesting pilot study (78) was conducted to determine the association between fat and oil intake and whole blood fatty acid profiles and to evaluate any association with markers of inflammation (PSA and CRP) and DNA damage in a group of 20 men with prostate cancer who accept to adhered to a Mediterranean style diet for 3 months. Volunteers were asked to eat 30–50 g of seeds and nuts daily as source of omega-3 polyunsaturated fatty acids (n3PUFA) and to take 15 mL or more of extra virgin olive oil as source of MUFA. Volunteers were asked to avoid the cooking of the oil to medium and high temperatures. They also were asked to reduce dairy intake to one portion daily and to reduce the intake of SFA by substituting butter and margarine with olive oil. Finally, volunteers were asked to eat no more than 400 g/week of red meat, which was substituted with oily fish at least once a week, and white meat, to avoid high temperature of cooking meat and fish, and to avoid intake of processed meats. After 3 months, authors observed a significant decrement of total SFA due to a significant decrease in stearic acid intake (*p* = 0.002). DHA and EPA showed a statistically significant increase in blood levels (*p* = 0.042), while arachidonic acid (AA) did not change significantly (*p* = 0.379). After 3 months, both the ratios of n6PUFA:n3PUFA and AA:EPA, expressed as mean percent, were decreased from baseline (4.7 vs 5.2, *p* = 0.019; 6.9 vs 8.58, *p* = 0.03, respectively). As regards C-reactive protein and PSA, authors did not find any significant change during the 3 months of the study; they observed a significant correlation between adherence to feeding based on the MD model and DNA damage. In particular, fish intake was protective vs DNA fragility (*r* = −0.452; *p* = 0.045) while dairy intake was significantly related with DNA fragility (*r* = 0.571; *p* = 0.008). Concerning the association between DNA damage, dietary fatty acid intake, and blood fatty acids, authors showed that intake of butter, cream, margarine, and red meat was directly associated with an increased DNA damage (*r* = 0.456; *p* = 0.043 and *r* = 0.576; *p* = 0.007, respectively). Authors also showed that MUFA and oleic acid intake had a protective role against DNA damage (*r* = −0.565, *p* = 0.009 and *r* = −0.514; *p* = 0.020, respectively); high blood levels of omega 6 polyunsaturated fatty acids (n6PUFA) intake and a high ratio of n6PUFA/n3PUFA were associated with an increased DNA damage (*r* = 0.536, *p* = 0.015 and *r* = 0.507, *p* = 0.023, respectively).

Subsequently (79), a second pilot study was conducted to evaluate the effect of 3 months of adherence to a dietary pattern based on the MD model on DNA damage and inflammation in a group of 20 men with diagnosed prostate cancer. Energy obtained from saturated fat decreased significantly (*p* < 0.001). Increases in intake of broccoli, sofrito (tomato sauce prepared with garlic and/or onion), pomegranate juice and green tea were statistically significant (*p* = 0.014, *p* = 0.006, *p* < 0.001 and *p* = 0.004, respectively); a decrease in refined carbohydrate intake was observed, by reduced intake of sweetened beverages and cakes or biscuits (*p* = 0.046 and *p* = 0.004, respectively). In addition, participants reduced the consumption of red meat (*p* < 0.001), and increased the consumption of fish (*p* < 0.001) and legumes (*p* = 0.005), so as not to change the amount of recruitment of the total protein (*p* = 0.149).

As observed by Bishop et al., there were no statistically significant relationships between high dietary adherence to the MD model and blood levels of C-reactive protein and PSA, either at baseline than after 3 months. After 3 months, following the dietary pattern based on the MD model was inversely associated with DNA damage (*p* = 0.013); particularly, MD model was protective against the peroxide-induced DNA damage (*p* = 0.009). Authors reported that consumption of green tea and intake of legumes were protective against DNA damage (*p* = 0.002; *p* = 0.004, respectively), while red meat intake was significantly associated with DNA damage (*p* = 0.007). A significant protective effect of vitamin C against DNA damage was observed at the end of the study (*p* = 0.007). In addition, protective effects of folate intake against hydrogen peroxide-induced DNA damage were observed after 3 months of dietary intervention (*p* = 0.023). Finally, the supposed protective effects due to an increased intake of vitamin E against basal and peroxide-induced DNA damage were not statistically significative at the end of the study (*p* = 0.175).

## Conclusion

The aim of our review was to analyze observational and case–control studies to point out the causative role of overweight, obesity, and dietary components on the cancer risk, particularly on risk of prostate cancer, and the effect of adherence to MD on the reduction of risk and mortality of prostate cancer. It is known that incidence and progression of cancer is multifactorial. Cancer of the large bowel, breast, endometrium, and prostate are also linked to a high BMI and to environmental factors, particularly low intake of vegetables and fruit, and high consumption of red and processed meat in feeding. Previous meta-analysis of prospective cohort studies suggested that high adherence to a diet based on the MD pattern gives a significant protection against overall mortality, and incidence of cancer (70). Epidemiological studies show that higher degree of adherence to the MD pattern is associated with a reduced mortality for cancer (67). Epidemiological studies also show that, especially in Western countries, approximately 25% of the incidence of colorectal cancer, 15% of breast cancer, and 10% of prostate, pancreas, and endometrial cancer can be prevented if we follow a diet based on the traditional MD pattern. Traditional MD is characterized by high consumption of vegetable foods, low consumption of red meat, and high consumption of olive oil (80). In our narrative review, we confirmed that higher degree of adherence to the traditional MD is associated with a reduction in total mortality, with respect to both deaths due to coronary heart disease and deaths due to cancer. Several studies provide evidence that nutrition is an important influencing factor for either tumor progression, recurrence, or survival; most of these reports investigated macronutrient composition or specific nutrients rather than dietary patterns. If the MD is dismantled into its components, it seems that there is no single ingredient or food category mediating any favorable effects. Protective effect is instead due to the whole food pattern characteristic for the MD. Protective effects of the MD might be due to several mechanisms, involving the suppression of spontaneous mutations, the regulation of the cell proliferation mechanisms, and the methylation of DNA and the induction of apoptosis. The main fat component of the MD is extra virgin olive oil, which is consumed in high amount by Mediterranean basin populations. Beneficial effects of olive oil are due to the monounsaturated fatty acid content, mainly oleic acid, and phenolic antioxidants contents, mainly phenols and flavonoids. The high content of oleic acid makes olive oil far less susceptible to oxidation than the polyunsaturated fatty acids. Phenolic compounds present in extra virgin olive oil have a protective role toward the oleic acid from the lipid peroxidation. Also, phenolic compounds exert some strong chemo-preventive effects, which are due to several mechanisms, including both direct antioxidant effects and actions on cancer cell signaling and cell cycle progression and proliferation. The protective effect of MD against cancer is also due to the high consumption of fruits and vegetables with a high content of flavonoids. The flavonoids exert multiple protective effects by inhibiting the inflammation and have a strong antioxidant activity. Flavonoids have anti-mutagenic and anti-proliferative properties involving cell signaling, cell cycle regulation, and angiogenesis. The protective effect of the MD against the prostate cancer is also due to the high consumption of tomato sauce. Lycopene is the most relevant functional component in tomatoes; is activated by the cooking of tomato sauce, which is used as a dressing, for example, of pizza or pasta. Lycopene exerts antioxidant properties by acting in the modulation of downregulation mechanisms of the inflammatory response. The beneficial effect of high intakes of vegetables and fruit, that are key features of the MD, is also because that their high consumption is associated to a very low intake of foods that are rich in SFA, and foods that are known to be associated with high risk of cancer, as red and processed meat. In conclusion, in this narrative review, we strongly restate how MD represents a healthy dietary pattern in the context of a healthy lifestyle habits. This review of the literature allows us to state emphatically how nutritional factors play an important role in the initiation and progression of cancer, including prostate cancer, and how a healthy dietary pattern represented by MD and its components, especially olive oil, could exert a protective role by the development of tumors, including prostate cancer.

## Author Contributions

All authors listed have made a substantial, direct, and intellectual contribution to the work and approved it for publication.

## Conflict of Interest Statement

The authors declare that the research was conducted in the absence of any commercial or financial relationships that could be construed as a potential conflict of interest.

## References

[1] GLOBOSCAN. (2012). Available from: http://www.wcrf.org/int/research-we-fund/continuous-update-project-findings-reports/prostate-cancer

[2] World Cancer Research Fund International/American Institute for Cancer Research Continuous Update Project Report. Diet, Nutrition, Physical Activity, and Prostate Cancer. (2014). 52 p. Available from: http://www.wcrf.org/int/research-we-fund/continuous-update-project-findings-reports/prostate-cancer

[3] Rossi S Crocetti E Capocaccia R Gatta G AIRTUM Working Group Estimates of cancer burden in Italy. Tumori (2013) 99:416–24. 10.1700/1334.14807 24158072

[4] Epstein JI Cubilla AL Humphrey PA Tumours of the prostate gland, seminal vesicles, penis, and scrotum. AFIP Atlas of Tumor Pathology (Vol. 14, Chap. 4). Washington, DC: ARP Press (2011).

[5] Humphrey PA Joachim Schüz J Cancers of the male reproductive organs. In: Stewart BW Wild CP, editors. World Cancer Report 2014. International Agency for Cancer Research (IARC) press (2014). p. 453–64. Available from: http://www.iarc.fr/en/publications/books/wcr/index.php

[6] Al Olama AA Kote-Jarai Z Berndt SI Conti DV Schumacher F Han Y A meta-analysis of 87,040 individuals identifies 23 new susceptibility loci for prostate cancer. Nat Genet (2014) 46:1103–9. 10.1038/ng.3094 25217961 PMC4383163

[7] Carruba G Cocciadiferro L Di Cristina A Granata OM Dolcemascolo C Campisi I Nutrition, aging and cancer: lessons from dietary intervention studies. Immun Ageing (2016) 13:13. 10.1186/s12979-016-0069-9 27057203 PMC4823849

[8] World Cancer Research Fund/American Institute for Cancer Research. Food, Nutrition, Physical Activity, and the Prevention of Cancer: A Global Perspective. Washington: American Institute for Cancer Research (2007). 517 p.

[9] Milner JA Molecular targets for bioactive food components. J Nutr (2004) 134:2492S–8S. 15333748 10.1093/jn/134.9.2492S

[10] Daniel M Tollefsbol TO. Epigenetic linkage of aging, cancer and nutrition. J Exp Biol (2015) 218:59–70. 10.1242/jeb.107110 25568452 PMC4286704

[11] Sofi F Macchi C Abbate R Gensini GF Casini A Mediterranean diet and health status: an updated meta-analysis and a proposal for a literature-based adherence score. Public Health Nutr (2013) 17:2769–82. 10.1017/S1368980013003169 24476641 PMC10282340

[12] Schwingshackl L Hoffmann G. Adherence to Mediterranean diet and risk of cancer: a systematic review and meta-analysis of observational studies. Int J Cancer (2014) 135:1884–97. 10.1002/ijc.28824 24599882

[13] Gronberg H. Prostate cancer epidemiology. Lancet (2003) 361:859–64. 10.1016/S0140-6736(03)12713-4 12642065

[14] Hsing AW Sakoda LC Chua S Jr Obesity, metabolic syndrome, and prostate cancer. Am J Clin Nutr (2007) 86:s843–57. 18265478 10.1093/ajcn/86.3.843S

[15] Allott EH Masko EM Freedland SJ. Obesity and prostate cancer: weighing the evidence. Eur Urol (2013) 63:800–9. 10.1016/j.eururo.2012.11.013 23219374 PMC3597763

[16] Calle EE Rodriguez C Walker-Thurmond K Thun MJ. Overweight, obesity, and mortality from cancer in a prospectively studied cohort of U.S. adults.N Engl J Med (2003) 348:1625–38. 10.1056/NEJMoa021423 12711737

[17] Renehan AG Tyson M Egger M Heller RF Zwahlen M Body mass index and incidence of cancer: a systematic review and meta-analysis of prospective observational studies. Lancet (2008) 371:569–78. 10.1016/S0140-6736(08)60269-X 18280327

[18] Bhaskaran K Douglas I Forbes H dos-Santos-Silva I Leon DA Smeeth L Body-mass index and risk of 22 specific cancers: a population-based cohort study of 5.24 million UK adults. Lancet (2014) 384:755–65. 10.1016/S0140-6736(14)60892-8 25129328 PMC4151483

[19] Bianchini F Kaaks R Vainio H. Overweight, obesity, and cancer risk. Lancet Oncol (2002) 3:565–74. 10.1016/S1470-2045(02)00849-5 12217794

[20] Hursting SD Dunlap SM. Obesity, metabolic dysregulation, and cancer: a growing concern and an inflammatory (and microenvironmental) issue. Ann N Y Acad Sci (2012) 1271:82–7. 10.1111/j.1749-6632.2012.06737.x 23050968 PMC3485672

[21] Platz EA Leitzmann MF Rifai N Kantoff PW Chen YC Stampfer MJ Sex steroid hormones and the androgen receptor gene CAG repeat and subsequent risk of prostate cancer in the prostate-specific antigen era. Cancer Epidemiol Biomarkers Prev (2005) 14:1262–9. 10.1158/1055-9965.EPI-04-0371 15894683

[22] Discacciati A Orsini N Andersson SO Andrén O Johansson JE Wolk A. Body mass index in early and middle-late adulthood and risk of localised, advanced and fatal prostate cancer: a population-based prospective study.Br J Cancer (2011) 105:1061–8. 10.1038/bjc.2011.319 21847119 PMC3185939

[23] Discacciati A Orsini N Wolk A Body mass index and incidence of localized and advanced prostate cancer – a dose–response metaanalysis of prospective studies. Ann Oncol (2012) 23:1665–71. 10.1093/annonc/mdr603 22228452

[24] Lawlor DA Harbord RM Sterne JA Timpson N Smith GD Mendelian randomization: using genes as instruments for making causal inferences in epidemiology. Stat Med (2008) 27:1133–63. 10.1002/sim.3235 17886233

[25] Smith GD Ebrahim S ‘Mendelian randomization’: can genetic epidemiology contribute to understanding environmental determinants of disease? Int J Epidemiol (2003) 32:1–22. 10.1093/ije/dyg070 12689998

[26] Davies NM Gaunt TR Lewis SJ Holly J Donovan JL Hamdy FC The effects of height and BMI on prostate cancer incidence and mortality: a Mendelian randomization study in 20,848 cases and 20,214 controls from the PRACTICAL consortium. Cancer Causes Control (2015) 26:1603–16. 10.1007/s10552-015-0654-9 26387087 PMC4596899

[27] Freedland SJ Platz EA. Obesity and prostate cancer: making sense out of apparently conflicting data. Epidemiol Rev (2007) 29:88–97. 10.1093/epirev/mxm006 17478439

[28] Lewis SJ Murad A Chen L Davey Smith G Donovan J Palmer T Associations between an obesity related genetic variant (FTO rs9939609) and prostate cancer risk. PLoS One (2010) 5:e13485. 10.1371/journal.pone.0013485 20976066 PMC2957440

[29] Benn M Tybjærg-Hansen A Smith GD Nordestgaard BG High body mass index and cancer risk – a Mendelian randomisation study. Eur J Epidemiol (2016) 31:879–92. 10.1007/s10654-016-0147-5 27061578

[30] Bouvard V Loomis D Guyton KZ Grosse Y Ghissassi FE Benbrahim-Tallaa L Carcinogenicity of consumption of red and processed meat. Lancet Oncol (2015) 16:1599–600. 10.1016/S1470-2045(15)00444-1 26514947

[31] Stacewicz-Sapuntzakis M Borthakur G Burns JL Bowen PE. Correlations of dietary patterns with prostate health. Mol Nutr Food Res (2008) 52:114–30. 10.1002/mnfr.200600296 18080240

[32] Kolonel LN Fat, meat, and prostate cancer. Epidemiol Rev (2001) 23:72–81. 10.1093/oxfordjournals.epirev.a000798 11588857

[33] Nowell S Ratnasinghe DL Ambrosone CB Williams S Teague-Ross T Trimble L Association of SULT1A1 phenotype and genotype with prostate cancer risk in African-Americans and Caucasians. Cancer Epidemiol Biomarkers Prev (2004) 13:270–6. 10.1158/1055-9965.EPI-03-0047 14973106

[34] Tavani A La Vecchia C Gallus S Lagiou P Trichopoulos D Levi F Red meat intake and cancer risk: a study in Italy. Int J Cancer (2000) 86:425–8. 10.1002/(SICI)1097-0215(20000501)86:3<425::AID-IJC19>3.0.CO;2-S 10760833

[35] Bosetti C Micelotta S Dal Maso L Talamini R Montella M Negri E Food groups and risk of prostate cancer in Italy. Int J Cancer (2004) 110:424–8. 10.1002/ijc.20142 15095309

[36] Cross AJ Peters U Kirsh VA Andriole GL Reding D Hayes RB A prospective study of meat and meat mutagens and prostate cancer risk. Cancer Res (2005) 65:11779–84. 10.1158/0008-5472.CAN-05-2191 16357191

[37] Sinha R Park Y Graubard BI Leitzmann MF Hollenbeck A Schatzkin A Meat and meat-related compounds and risk of prostate cancer in a large prospective cohort study in the United States. Am J Epidemiol (2009) 170:1165–77. 10.1093/aje/kwp280 19808637 PMC2781742

[38] Wu K Spiegelman D Hou T Albanes D Allen NE Berndt SI Associations between unprocessed red and processed meat, poultry, seafood and egg intake and the risk of prostate cancer: a pooled analysis of 15 prospective cohort studies. Int J Cancer (2016) 138:2368–82. 10.1002/ijc.29973 26685908 PMC4837898

[39] Gilsing AM Weijenberg MP Goldbohm RA Dagnelie PC van den Brandt PA Schouten LJ. Vegetarianism, low meat consumption and the risk of lung, postmenopausal breast and prostate cancer in a population-based cohort study. Eur J Clin Nutr (2016) 70:723–9. 10.1038/ejcn.2016.25 26931668

[40] McCullough ML Giovannucci EL Diet and cancer prevention. Oncogene (2004) 23:6349–64. 10.1038/sj.onc.1207716 15322510

[41] Kooiman GG Martin FL Williams JA Grover PL Phillips DH Muir GH. The influence of dietary and environmental factors on prostate cancer risk. Prostate Cancer Prostatic Dis (2000) 3:256–8. 10.1038/sj.pcan.4500489 12497073

[42] Giovannucci E Rimm EB Colditz GA Stampfer MJ Ascherio A Chute CG A prospective study of dietary fat and risk of prostate cancer. J Natl Cancer Inst (1993) 85:1571–9. 10.1093/jnci/85.19.1571 8105097

[43] Gann PH Hennekens CH Sacks FM Grodstein F Giovannucci EL Stampfer MJ. Prospective study of plasma fatty acids and risk of prostate cancer. J Natl Cancer Inst (1994) 86:281–6. 10.1093/jnci/86.4.281 8158682

[44] Simon JA Chen Y Bentet S The relation of α-linolenic acid to the risk of prostate cancer: a systematic review and meta-analysis. Am J Clin Nutr (2009) 89(Suppl):1558S–64S. 10.3945/ajcn.2009.26736E 19321563

[45] Szymanski KM Wheeler DC Mucci LA. Fish consumption and prostate cancer risk: a review and meta-analysis. Am J Clin Nutr (2010) 92:1223–33. 10.3945/ajcn.2010.29530 20844069

[46] Kurahashi N Inoue M Iwasaki M Sasazuki S Tsugane AS Japan Public Health Center-Based Prospective Study Group. Dairy product, saturated fatty acid, and calcium intake and prostate cancer in a prospective cohort of Japanese men. Cancer Epidemiol Biomarkers Prev (2008) 17:930–7. 10.1158/1055-9965.EPI-07-2681 18398033

[47] Song Y Chavarro JE Cao Y Qiu W Mucci L Sesso HD Whole milk intake is associated with prostate cancer-specific mortality among U.S. male physicians. J Nutr (2013) 143:189–96. 10.3945/jn.112.168484 23256145 PMC3542910

[48] Abid Z Cross AJ Sinha R. Meat, dairy, and cancer. Am J Clin Nutr (2014) 100(Suppl):386S–93S. 10.3945/ajcn.113.071597 24847855 PMC4144110

[49] Park Y Leitzmann MF Subar AF Hollenbeck A Schatzkin A. Dairy food, calcium, and risk of cancer in the NIH-AARP diet and health study. Arch Intern Med (2009) 169:391–401. 10.1001/archinternmed.2008.578 19237724 PMC2796799

[50] Huncharek M Muscat J Kupelnick B. Dairy products, dietary calcium and vitamin D intake as risk factors for prostate cancer: a meta-analysis of 26,769 cases from 45 observational studies. Nutr Cancer (2008) 60:421–41. 10.1080/01635580801911779 18584476

[51] Bernichtein S Pigat N Capiod T Boutillon F Verkarre V Camparo P High milk consumption does not affect prostate tumor progression in two mouse models of benign and neoplastic lesions. PLoS One (2015) 10:e0125423 10.1371/journal.pone.0125423 25938513 PMC4418739

[52] Aune D Navarro Rosenblatt DA Chan DS Vieira AR Vieira R Greenwood DC Dairy products, calcium, and prostate cancer risk: a systematic review and meta-analysis of cohort studies. Am J Clin Nutr (2015) 101:87–117. 10.3945/ajcn.113.067157 25527754

[53] Rodriguez C McCullough ML Mondul AM Jacobs EJ Fakhrabadi-Shokoohi D Giovannucci EL Calcium, dairy products, and risk of prostate cancer in a prospective cohort of United States men. Cancer Epidemiol Biomarkers Prev (2003) 12:597–603. 12869397

[54] Xu Y Shao X Yao Y Xu L Chang L Jiang Z Positive association between circulating 25-hydroxyvitamin D levels and prostate cancer risk: new findings from an updated meta-analysis. J Cancer Res Clin Oncol (2014) 140:1465–77. 10.1007/s00432-014-1706-3 24838848 PMC11823905

[55] Jacobs ET Kohler LN Kunihiro AG Jurutka PW. Vitamin D and colorectal, breast, and prostate cancers: a review of the epidemiological evidence. J Cancer (2016) 7:232–40. 10.7150/jca.13403 26918035 PMC4747876

[56] Willett WC Sacks F Trichopoulou A Drescher G Ferro-Luzzi A Helsing E Mediterranean diet pyramid: a cultural model for healthy eating. Am J Clin Nutr (1995) 61(Suppl 6):S1402–6. 7754995 10.1093/ajcn/61.6.1402S

[57] Fundación Dieta Mediterránea. (2010). Available from: https://dietamediterranea.com

[58] Keys AB Seven Countries: A Multivariate Analysis of Death and Coronary Heart Disease. Cambridge, MA: Harvard University Press (1980). 381 p.

[59] Schröder H Marrugat J Vila J Covas MI Elosua R Adherence to the traditional Mediterranean diet is inversely associated with body mass index and obesity in a spanish population. J Nutr (2004) 134:3355–61. 15570037 10.1093/jn/134.12.3355

[60] Trichopoulou A Naska A Orfanos P Trichopoulos D Mediterranean diet in relation to body mass index and waist-to-hip ratio: the Greek European Prospective Investigation into Cancer and Nutrition Study. Am J Clin Nutr (2005) 82:935–40. 16280422 10.1093/ajcn/82.5.935

[61] Sànchez-Villegas A Bes-Rastrollo M Martìnez-Gonzàlez MA Serra-Majem L Adherence to a Mediterranean dietary pattern and weight gain in a follow-up study: the SUN cohort. Int J Obes (2006) 30:350–8. 10.1038/sj.ijo.0803118 16231028

[62] Rossi M Negri E Bosetti C Dal Maso L Talamini R Giacosa A Mediterranean diet in relation to body mass index and waist-to-hip ratio. Public Health Nutr (2008) 11(2):214–7. 10.1017/S1368980007000833 17686205

[63] Shai I Schwarzfuchs D Henkin Y Shahar DR Witkow S Greenberg I Weight loss with a low-carbohydrate, Mediterranean, or low-fat diet. N Engl J Med (2008) 359:229–41. 10.1056/NEJMoa0708681 18635428

[64] Ajala O English P Pinkney J. Systematic review and meta-analysis of different dietary approaches to the management of type 2 diabetes. Am J Clin Nutr (2013) 97:505–16. 10.3945/ajcn.112.042457 23364002

[65] Huo R Du T Xu Y Xu W Chen X Sun K Effects of Mediterranean-style diet on glycemic control, weight loss and cardiovascular risk factors among type 2 diabetes individuals: a meta-analysis. Eur J Clin Nutr (2015) 69:1200–8. 10.1038/ejcn.2014.243 25369829

[66] Lasa A Miranda J Bulló M Casas R Salas-Salvadó J Larretxi I Comparative effect of two Mediterranean diets versus a low-fat diet on glycaemic control in individuals with type 2 diabetes. Eur J Clin Nutr (2014) 68(7):767–72. 10.1038/ejcn.2014.1 24518752

[67] Trichopoulou A Costacou T Bamia C Trichopoulos D Adherence to a Mediterranean diet and survival in a Greek population. N Engl J Med (2003) 348:2599–608. 10.1056/NEJMoa025039 12826634

[68] Trichopoulou A Kouris-Blazos A Mark L Wahlqvist ML Diet and overall survival in elderly people. BMJ (1995) 311:1457 10.1136/bmj.311.7018.1457 8520331 PMC2543726

[69] Turati F Rossi M Pelucchi C Levi F La Vecchia C. Fruit and vegetables and cancer risk: a review of southern European studies. Br J Nutr (2015) 113:S102–10. 10.1017/S0007114515000148 26148912

[70] Sofi F Cesari F Abbate R Gensini GF Casini A. Adherence to Mediterranean diet and health status: meta-analysis. BMJ (2008) 337:a1344. 10.1136/bmj.a1344 18786971 PMC2533524

[71] Sofi F Abbate R Gensini GF Casini A. Accruing evidence on benefits of adherence to the Mediterranean diet on health: an updated systematic review and meta-analysis. Am J Clin Nutr (2010) 92:1189–96. 10.3945/ajcn.2010.29673 20810976

[72] Psaltopoulou T Kosti RI Haidopoulos D Dimopoulos M Panagiotakos DB Olive oil intake is inversely related to cancer prevalence: a systematic review and a metaanalysis of 13800 patients and 23340 controls in 19 observational studies. Lipids Health Dis (2011) 10:127 10.1186/1476-511X-10-127 21801436 PMC3199852

[73] Tzonou A Signorello LB Lagiou P Wuu J Trichopoulos D Trichopoulou A. Diet and cancer of the prostate: a case-control study in Greece. Int J Cancer (1999) 80:704–8. 10.1002/(SICI)1097-0215(19990301)80:5<704::AID-IJC13>3.0.CO;2-Z 10048971

[74] Sotiroudis TG Kyrtopoulos SA. Anticarcinogenic compounds of olive oil and related biomarkers. Eur J Nutr (2008) 47(Suppl 2):69–72. 10.1007/s00394-008-2008-9 18458836

[75] Key TJ Appleby PN Travis RC Albanes D Alberg AJ Barricarte A Carotenoids, retinol, tocopherols, and prostate cancer risk: pooled analysis of 15 studies. Am J Clin Nutr (2015) 102:1142–57. 10.3945/ajcn.115.114306 26447150 PMC4625592

[76] Fielding JM Rowley KG Kooper P O’Dea K. Increases in plasma lycopene concentration after consumption of tomatoes cooked with olive oil. Asia Pac J Clin Nutr (2005) 14:131–6. 15927929

[77] Gann PH Ma J Giovannucci E Willett W Sacks FM Hennekens CH Lower prostate cancer risk in men with elevated plasma lycopene levels: results of a prospective analysis. Cancer Res (1999) 59:1225–30. 10096552

[78] Bishop KS Erdrich S Karunasinghe N Han DY Zhu S Jesuthasan A An investigation into the association between DNA damage and dietary fatty acid in men with prostate cancer. Nutrients (2015) 7:405–22. 10.3390/nu7010405 25580814 PMC4303847

[79] Erdrich S Bishop KS Karunasinghe N Han DY Ferguson LR. A pilot study to investigate if New Zealand men with prostate cancer benefit from a Mediterranean-style diet. PeerJ (2015) 3:e1080. 10.7717/peerj.1080 26157638 PMC4493678

[80] Trichopoulou A Lagiou P Kuper H Trichopoulos D. Cancer and Mediterranean dietary traditions. Cancer Epidemiol Biomarkers Prev (2000) 9:869–73. 11008902

